# Thalassaemia and Aberrations of Growth and Puberty

**DOI:** 10.4084/MJHID.2009.003

**Published:** 2009-07-27

**Authors:** Andreas Kyriakou, Nicos Skordis

**Affiliations:** Pediatric Endocrine Unit, Dept. of Pediatrics, Makarios Hospital, Nicosia, Cyprus

## Abstract

Endocrine dysfunction in Thalassaemia major (TM) is a common and disturbing complication, which requires prompt recognition and treatment. The contribution of the underlying molecular defect in TM to the development of endocrinopathies is significant because the patients with the more severe genetic defects have a greater rate of iron loading through higher red cell consumption. TM patients frequently present delay of growth and puberty with reduction of final height. The pathogenesis of growth failure is multifactorial and is mainly due to chronic anemia and hypoxia, chronic liver disease, zinc and folic acid deficiency, iron overload, intensive use of chelating agents, emotional factors, and endocrinopathies (hypogonadism, delayed puberty, hypothyroidism) and GH-IGF-1 axis dysregulation. Although appropriate iron chelation therapy can improve growth and development, TM children and adolescents treated intensively with desferrioxamine remain short as well, showing body disproportion between the upper and lower body segment. Body disproportion is independent of pubertal or prepubertal period of greater height gain. Treatment with recombinant GH (rhGH) is recommended when GH deficiency is established, and even so, the therapeutic response is often non satisfactory. Growth acceleration is mostly promoted with sex steroids in children with associated pubertal delay. Sexual complications in TM, which include Delayed Puberty, Arrested Puberty and Hypogonadism, present the commonest endocrine complication. Iron deposition on gonadotroph cells of the pituitary leads to disruption of gonadotrophin production which is proven by the poor response of FSH and LH to GnRH stimulation. In the majority of patients gonadal function is normal as most women with Amenorrhea are capable of achieving pregnancy with hormonal treatment and similarly men with azoospermia become fathers. Secondary Hypogonadism appears later in life, and is manifested in women as Secondary Amenorrhea and in men as decline in sexual drive and azzoospermia. The damage to the hypothalamus and pituitary is progressive, even when intensive chelating therapy is given and the appearance of Hypogonadism in both sexes is often unavoidable. Close follow up and proper management is crucial for every patient with TM. Early recognition of growth disturbance and prevention of hypogonadism by early and judicious chelation therapy is mandatory for the improvement of their quality of life. Patients with TM can now live a better life due to modern advances in their medical care and our better understanding in the pathogenesis, manifestation and prevention of endocrine complications.

## Preface:

Treatment of beta-Thalassaemia Major (TM) is based on regular blood transfusions to maintain the pre-transfusional Hb level above 9 gr/dl and appropriate chelation therapy to avoid the consequences of iron overload. The metabolically active iron catalyses the formation of free radicals, which damage membrane lipids leading to cell death and eventually organ failure. The endocrine glands are particularly vulnerable to the excess iron, so that the appearance of endocrine dysfunction in Thalassaemia major is a common and disturbing complication, which requires prompt recognition and treatment. The contribution of the underlying molecular defect in TM to the development of endocrinopathies and particularly Hypogonadotrophic Hypogonadism is significant because the patients with the more severe genetic defects have a greater rate of iron loading through higher red cell consumption.^1^

## Growth:

TM patients frequently present delay of growth and puberty with reduction of final height. Growth failure in TM has been recognised for many years, and has persisted despite major treatment advances. The child with TM has a particular growth pattern, which is relatively normal until age 9–10 years; after this age a slowing down of growth velocity and a reduced or absent pubertal growth spurt are observed. The growth plate fusion is usually delayed until the end of the second decade of life.^2^ The pathogenesis of growth failure is multifactorial^3^,^4^ and is mainly due to chronic anemia and hypoxia, chronic liver disease, zinc and folic acid deficiency, iron overload, intensive use of chelating agents, emotional factors, endocrinepathies (hypogonadism, delayed puberty, hypothyroidism) and GH-IGF-1 axis dysregulation.

Chronic hypoxia is no longer a contributing factor nowadays in properly treated children. Linear growth in childhood is disrupted only in a small percentage of children due to anemia, ineffective erythropoiesis and iron overload. During the first decade of life the maintenance of haemoglobin levels above 9 g/dL together with adequate iron chelation therapy makes the children with TM indistinguishable from their non thalassaemic peers.^5^,^6^

Zinc deficiency is probably a concomitant factor in growth failure. The effects of zinc supplementation on growth velocity were assessed in 22 patients with biochemical evidence of zinc deficiency. The mean height velocity of zinc supplemented children was significantly greater that of normal children^7^. The crucial role of zinc deficiency however was not confirmed by other studies.^8^,^9^

It is well known that high serum ferritin levels during the first decade of life are associated with final short stature^10^,^11^, indicating that appropriate iron chelation therapy can prevent or limit this complication. However, several studies showed high prevalence of short stature in TM children and adolescence treated intensively with desferrioxamine (DFX).^4^,^12^,^13^ In addition, premature chelating therapy, between ages of 2–5 years, may have deleterious effects on growth.^12^,^14^,^15^ DFX is proven to inhibit cell proliferation, DNA synthesis, collagen formation and trace mineral deposition such as cooper and zinc. Mineral depletion may result in a decrease of alkaline phosphatase activity. This complex mechanism results in platyspondylosis with flattening of the vertebral bodies and consequent shortening of the spinal height, resulting in truncal shortening, also in the presence of normal stature.^1^,^2^,^15^–^20^

**Figure 1.**
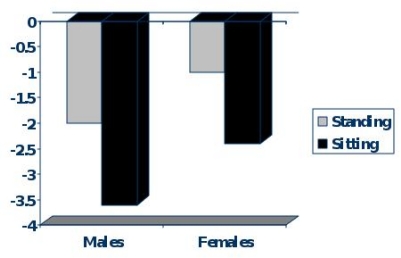
Standing and Sitting Final Height SDS in adult patients with Thalassaemia of both sexes.

**Figure 2.**
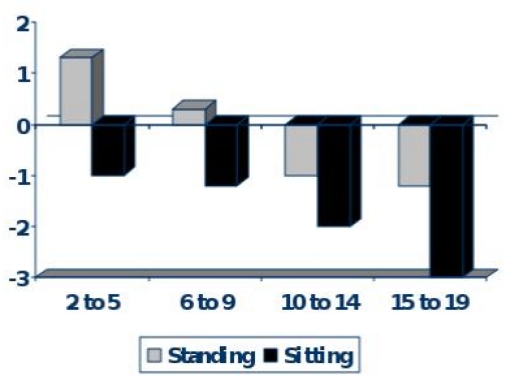
Standing and Sitting Height SDS in different age groups in children with Thalassaemia of both sexes.

Body disproportion between the upper (short) and lower body segment (normal) is observed in approximately 15–40% of TM patients.^18^ Spinal growth impairment starts during infancy and deteriorates progressively. Most Thalassaemics, especially males do not reach their target height and their Sitting height is more severely affected (Figure 1). During puberty hypogonadism further impairs spinal growth (Figure 2).

Sex steroids replacement therapy cannot adversely affect body disproportion, as no difference has been observed in pubertal growth and final height between treated Hypogonadal patients compared to those with spontaneous puberty and truncal shortening at final height is evident in patients with either spontaneous or induced puberty.^17^ Body disproportion therefore is independent of pubertal or prepubertal period of greater height gain. Decrease in upper/lower segment ratio has been reported in patients who have been poorly chelated during childhood and adolescence, so that other contributing factors besides chelation therapy or the disease itself influence spinal growth.^21^

The hormonal cause of growth retardation in TM children is complex. Besides hypogonadism and hypothyroidism it has been apparent that GH-IGF-1 axis plays a role in their abnormal growth.^22^ Evaluation of GH-IGF-1 axis has given contradictory results. The response of GH to stimulation tests has been found to be normal^23^,^24^ or reduced with a variable prevalence^4^,^25^–^33^ in a number of TM patients with short stature. De Sanctis *et al* reported 3.1% of thalassemics to have GHRH-GH-IGF axis dysfunction.^21^ The presence of GH neurosecretory dysfunction is supported by the impaired 24 hour GH secretion^29^,^34^–^37^ due to hypothalamic or pituitary dysfunction. The anterior pituitary is particularly sensitive to free radical oxidative stress. MRI of pituitary shows that even a modest amount of iron deposition within the anterior pituitary can interfere with its function^38^. Sex hormones play an important role in pubertal growth spurt and TM patients with delayed puberty do not exhibit normal growth spurt; their GH peak amplitude is reduced as well as their nocturnal GH levels.^26^,^34^,^39^ Several authors have reported normal GH and GHBP levels but low levels of IGF-1 and IGFBP-3 which are not properly increased with IGF-1 generation test, suggesting that insensitivity to GH action may be the cause of abnormal growth.^20^,^25^,^28^,^32^,^40^–^42^ However, a lack of correlation between IGF-1, IGFBP-3 and height SDS in TM children with growth failure may indicate that growth failure is not specifically related to GH-IGF-1 axis. There is a possibility that DFX and iron loading at the growth plate have a deleterious effect in local IGF-1 production and growth regulation.^43^,^44^

Can children with TM attain normal stature and develop normally with early and reasonable DFX treatment? Although iron chelation can decrease the frequency of endocrinopathies, early DFX treatment may result in growth impairment. On the other hand poor compliance with DFX may eventually lead to severe iron burden, gonadal dysfunction and eventually growth failure. The benefits of treatment should be weighted against the potential adverse effects and the caring physician should balance between the efficacy and the injudicious use of DFX. An ideal therapeutic regimen, which will avoid the toxic effects of iron overload and that of continuous subcutaneous chelation therapy, has yet to be found. It is therefore recommended that growth in both standing and sitting position should be assessed at 6-month intervals in order to detect early growth failure. Long-term observations on the effect of therapy are needed before this mysterious puzzle is solved. Alternative oral chelation agents are often an option in cases of DFX toxicity, although some bone lesions remain irreversible. Prevention of growth retardation is essential. Monitoring growth in all children by using growth charts for both standing and sitting height is mandatory. The mean hemoglobin levels must be kept near 9 gr/dl. Prompt initiation of iron chelation therapy prevents pituitary haemosiderosis, which is the main cause of GH insufficiency.

Treatment with recombinant GH (rhGH) is recommended when GH deficiency is established. Therapeutic response with rhGH administration in cases with GH deficiency, is often non satisfactory. In poor responders such treatment should be discontinued. Treatment with rhGH for 1 year seems to be effective in increasing growth velocity without causing adverse effects on bone maturation, glucose tolerance, serum lipids and blood pressure.^45^–^52^ The encouraging results described during the first year of rhGH treatment do not persist during the second and the third years. This is because increase in bone age with continued treatment is equal to or slightly greater than the height age increase^49^. Prolonged therapy with rhGH could not improve final height; on the contrary a negative effect may be hypothesized^52^. Growth acceleration is mostly promoted with sex steroids in children with pubertal delay as sexual complications present a significant issue in Thalassaemics.

## Protocol for investigation of Thalassaemic children:

1. Measure current height both standing and sitting and plot on the growth chart. Calculate the target height based on parental heights. Compare with previous measurement to estimate the growth velocity. Examine pubertal status. Note any physical disproportion. Review emotional and social status.
2. Assess bone maturation
3. Routine blood tests including liver function tests, ferritin, biochemical profile, and zinc
4. Urine analysis
5. Thyroid function tests (Free T4, TSH)

IFG-1 and IGFBP-3

Stimulation tests to assess GH secretion, where at least two tests are required. Priming with sex steroids (testosterone depot 100 mg IM in boys and Ethinyl Estradiol 10 mcg orally for 3 days in girls 72hrs before test may be necessary in children who are prepubertal and have a bone age of 10 years.

IGF-1 generation test in patients with low levels of IFG-1 and IFGBP-3 and normal GH secretion to exclude GH resistance.

**Puberty** is the period of life that leads to adulthood through dramatic physiologic and psychologic changes. It is the period during which physical and hormonal changes occur such that the capability of sexual reproduction is attained. This biological phenomenon, which is the result of the activation of the Hypothalamic – Pituitary – Gonadal axis and clinically manifested by the appearance of sexual characteristics comprises this unique and integrated transition from childhood to young adulthood.

**Figure 3.**
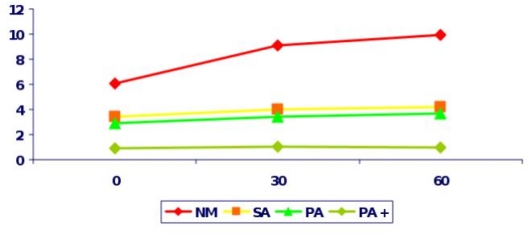
FSH levels after GnRH stimulation in Thalassaemic women with Normal menstrual cycles (NM), Primary Amenorrhea (PA), Primary Amenorrhea with additional endocrinopathies (PA+) and Secondary Amenorrhea (SA).X-axis: time in minutes, y-axis: FSH in miu/l

**Figure 4.**
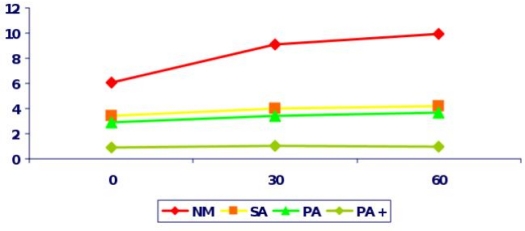
LH levels after GnRH stimulation in Thalassaemic women with Normal menstrual cycles (NM), primary Amenorrhea (PA), Primary Amenorrhea with additional endocrinopathies (PA+) and Secondary Amenorrhea (SA). - x-axis xtime in minutes, y-axis:LH in miu/l

Sexual complications in TM present the commonest endocrine complication in almost all studies (Table 1). These include: Delayed Puberty, Arrested Puberty and Hypogonadism. Delayed puberty is defined as the absence of any pubertal sign in girls (breast enlargement) and in boys (testicular enlargement) by the age of 13 and 14 years respectively. Delayed puberty in TM is almost always due to Hypogonadotrophic Hypogonadism, which still remains the most stressful complication.^53^ Iron deposition on gonadotrophic cells of the pituitary leads to disruption of gonadotrophin production which is proven by the poor response of FSH and LH to GnRH stimulation and clinically manifested as Hypogonadotrophic Hypogonadism (Figures 3, 4). Women have Primary Amenorrhea and additional endocrinopathies have a completely blunted response to GnRH test indicating the severity of haemosiderosis. Anterior pituitary function correlates well with tissue iron deposition in the pituitary gland, as quantitatively determined by MRI measurements (T2*).^38^,^54^ However, there is no correlation between MRI measurements, the GnRH stimulation test and the clinical status of the patients^38^.

The association of susceptibility to develop Hypogonadotrophic Hypogonadism with the genotype has already been proven.^1^,^55^,^56^ The contribution of the underlying molecular defect in TM to the development of endocrinopathies in TM and particularly Hypogonatotrophic Hypogonadism is significant, because the patients with the more severe defects have a greater rate of iron loading through higher red cell consumption and probably a different vulnerability to free radical damage.^1^,^55^

Hypogonatotrophic hypogonadism in Thalassaemia is related not only to iron toxicity on gonadotroph cells but also to iron toxicity on the adipose tissue thus changing the physiological role of leptin in sexual maturation and fertility. Leptin is a polypeptide hormone that is produced in fat cells due to the expression of the ob gene. In girls leptin levels increase dramatically as puberty develops and stimulate the Hypothalamic-Pituitary-Gonadal axis.^57^,^58^

There is evidence that this hormone acts as a permissive signal allowing puberty to precede. The impaired synthesis of leptin in Thalassaemic patients seems to be related to transferrin receptor levels and therefore iron toxicity but further research will be required to elucidate this critical issue^59^,^60^. Gonadal iron deposition occasionally occurs. The iron deposition on the gonads is a rarer condition. In the majority of patients gonadal function is normal as most women with Amenorrhea are capable of ovulating with hormonal treatment and similarly men with azoospermia become fathers.

Arrested puberty is defined as the absence of further pubertal progression -once puberty has started -for more than one year, where testicular volume in boys never exists 6 to 8 ml and breast size in girls remains unchanged. Failure of sexual development by the age of 15 to 16 years in both sexes is defined as Hypogonadism. Secondary Hypogonadism appears later in life, and is manifested in women as Secondary Amenorrhea and in men as decline in sexual drive and azzoospermia.

Adolescent girls with TM often present with Primary Amenorrhea and boys fail to become well virilized. The damage to the hypothalamus and pituitary is progressive, even when intensive chelating therapy is given and the appearance of Hypogonadism in both sexes is often unavoidable^1^,^55^,^61^. Most women with TM manifest Secondary Amenorrhea at some stage in their life and men develop hypogonadism in their 3^rd^ decade after being normal for some years and even becoming fathers.^55^,^62^,^63^

The overall frequency of bone disease in hypogonadal patients with TM is increased compared with those of normal gonadal function^1^,^64^. Sex steroids regulate skeletal maturation and preservation in both men and women, therefore the impact of gonadal insufficiency on skeletal integrity has been widely recognised in both genders. BMD normally rises at a steady rate throughout childhood until around the age of 12, and then there is a sudden acceleration of bone mineral accretion which coincides with the onset of puberty and the pubertal growth spurt. Failure to progress normally through puberty is associated with failure of achievement of peak bone mass, which is a contributing factor to the ultimate bone disease in Thalassaemia.^44^,^65^,^66^

## Protocol for investigation of pubertal disorder:

The absence of any clinical pubertal signs in a boy (testicular enlargement) older that 14 years and in a girl (breast development) older than 13 years requires investigation.

Measure Testosterone in the boy and Oestradiol in the girl. DHEA-S in both sexes is often helpful

Perform the GnRH test to evaluate the pituitary capacity to secrete the gonadotropins FSH and LH, where the response in Hypogonadism is low

Bone age is helpful for the treatment decision options

**Therapeutic approach** in delayed puberty should mimic biological and biochemical pubertal changes, aiming on promotion of linear growth as well.^67^–^70^

Induction of puberty in boys can be achieved with Testosterone depot IM 25–50mg monthly for 6 months and reassessment. Pubic hair will appear and penile size will increase. Increase in testicular volume indicates activation of the axis and release of Gonadotrophins (FSH and LH), where no further treatment is needed except for close observation. In case where testicular size is unchanged, then treatment is continued for 6 months and subsequently the dose is increased to 100 mg monthly for one year. Therapeutic schedule is determined by the growth potential, clinical response and emotional factors. For testicular enlargement, the therapeutic regime is altered to the combination of hCG and hMG or recFSH, both of which mimic the pituitary Gonadotrophins. The final adult dose of Testosterone depot, is always individualized and usually 50mg/weekly IM or alternatively transdermally in patches 5 mg/daily. The oral route (testosterone undeconate) should be avoided due to liver toxicity.

For pubertal induction in girls oral Ethinylestradiol is preferred at the dose 100 ng/kg/d for 6 months, where increase in breast size and growth acceleration is noted. This dose is continued for additional 6 months and increased to 200 ng/kg/d for the subsequent year. Therapeutic schedule is determined by the same factors as in boys. The adult dose is 400 ng/kg/d, where the uterine size is satisfactorily increased for the induction of menarche. Induction of puberty can be successfully achieved by the transdermal use of Estrogens.

Menarche is achieved by the addition of Medroxyprogesterone 10 mg/d for 10 days when the size of the uterus exceeds 5 cm. When menstrual bleeding occurs spontaneously during Estrogen treatment, the regime should be adjusted. For maintenance of the menstrual cycle the use of Estrogens (Conjugated Estrogens 0.625 ή 1.25 mg, Ethinyl Estradiol 20 μg) from day 1^st^ to 25^th^ and Progesterone from day 14^th^ to 25^th^ is required. The transdermal use of Estradiol και Norethisterone is advantageous due to decreased liver toxicity and preferred in most cases.

Normal sexual activity and reproductive capacity have become demanding tasks for women with TM. Despite the presence of Hypogonadotrophic Hypogonadism and severe iron deposition, ovarian function may be preserved, as they are still able to increase oestradiol level following gonadotrophin stimulation test and produce ova.^71^,^72^ Women with TM who are regularly transfused and are well chelated become able to conceive after a closely monitored treatment.^63^,^73^ Males who have normal gonadal function maintain their spermatogenic ability and therefore, frequently become fathers. On the other side of the spectrum, in cases where impaired spermatogenesis is present, a combination treatment with hCG and hMG/ recFSH has proven to be beneficial in improving their reproductive capacity.^70^

## Epilogue:

Several endocrine glands may be affected in patients with TM in childhood, adolescence and adulthood. Pituitary damage due to iron overload is the underlying pathogenetic factor in hypogonadism and partly contributes to poor growth. Luckily these complications do not adversely affect the life expectancy of these individuals. Close follow up and proper management is crucial for every patient. Early recognition of growth disturbance and prevention of hypogonadism by early and judicious chelation therapy is mandatory for the improvement of their quality of life. Patients with TM can now live a better life due to modern advances in their medical care and our better understanding in the pathogenesis, manifestation and prevention of endocrine complications.

## Figures and Tables

**Table 1:** Prevalence of endocrine complications in patients with Thalassaemia in Cyprus, other countries and in the TIF multicenter study.^30^

**Study**	**Cyprus**	**Greece 199574**	**Italy 199553**	**N. America 200475**	**TIF 200430**
N^o^ of patients	435	262	1861	342	3817
Hypogonadism	32.5	42	49	35	40.5
Short Stature	35	32	----	----	30.8
Short Sitting height	72	----	----	----	----
Hypothyroidism	5.9	4	6.2	9	3.2
Hypoparathyroidism	1.2	4	3.6	4	6.9
DM/IGT	9.4	5/27	4.9	10	3.2/6.5
